# Hierarchical Self-Assembled Structures from Diblock Copolymer Mixtures by Competitive Hydrogen Bonding Strength

**DOI:** 10.3390/molecules23092242

**Published:** 2018-09-03

**Authors:** Tzu-Chun Tseng, Shiao-Wei Kuo

**Affiliations:** 1 Department of Materials and Optoelectronic Science, Center for Functional Polymers and Supramolecular Materials, National Sun Yat-Sen University, Kaohsiung 80424, Taiwan; mix50832@gmail.com; 2 Department of Medicinal and Applied Chemistry, Kaohsiung Medical University, Kaohsiung 80424, Taiwan

**Keywords:** self-assembly, hydrogen bonding, block copolymers, living polymerization

## Abstract

In this work we prepared poly(styrene–*b*–vinylphenol) (PS-*b*-PVPh) by sequential anionic living polymerization and poly(ethylene oxide-*b*-4-vinylpyridine) (PEO-*b*-P4VP) by reversible addition fragmentation chain transfer polymerization (RAFT) by using poly(ethylene oxide) 4-cyano-4-(phenylcarbonothioylthio)pentanoate (PEO-SC(S)Ph) as a macroinitiator with two hydrogen bonded acceptor groups. When blending with disordered PEO-*b*-P4VP diblock copolymer, we found the order-order self-assembled structure transition from lamellar structure for pure PS-*b*-PVPh to cylindrical, worm-like, and finally to PEO crystalline lamellar structures. Taking the advantage of the Δ*K* effect from competitive hydrogen bonding strengths between PVPh/P4VP and PVPh/PEO domains, it could form the hierarchical self-assembled morphologies such as core–shell cylindrical nanostructure.

## 1. Introduction

Diblock copolymers can display different self-assembly behaviors, including lamellar, gyroid, cylinder, and spherical nanostructures, which possess several potential applications in drug delivery, photonic crystals, and nanotechnology [1,2,3,4]. However, mediating the different molecular weights of each block segment to control the volume fraction by using living polymerization methods may be time-consuming and difficult and thus blending the homopolymer or another block copolymer through intermolecular hydrogen bonding interactions has received much interest recently [5,6,7]. For diblock copolymer/homopolymer (A-*b*-B/C) blends with hydrogen bonding interactions, there are four different situations that have been proposed based on experimental and theoretical results [8,9,10,11,12,13,14,15,16,17,18,19,20,21,22,23,24,25]. However, only the typical self-assembly nanostructures as diblock copolymers were observed for A-*b*-B/C blends including PS-*b*-PVPh blending with poly(methyl methacrylate) (PMMA), polycaprolactone (PCL), PEO, P2VP, and P4VP homopolymers; PS-*b*-P2VP, and PCL-*b*-P4VP diblock copolymers blending PVPh homopolymer; PVPh-*b*-PCL or PVPh-*b*-PMMA blending with PVP homopolymer [8,9,10,11,12,13,14,15,16,17,18,19,20,21,22,23,24,25].

Furthermore, extending the A-*b*-B/C mixtures to A-*b*-B/C-*b*-D diblock copolymer mixtures as similar triblock copolymer has also received much interest recently since they provide a simpler approach for the formation of hierarchical self-assembled structures such as three lamellae or core–shell cylinder structures [26,27,28,29,30,31,32,33,34,35,36,37,38]. In general, the B and C blocks would be miscible through hydrogen bonding interactions, but the A and D blocks are immiscible with each other. For example, Matsushita and we have found three phase nanostructures either in bulk or in solution state for PS-*b*-PVPh blended with P4VP-*b*-PCL, P4VP-*b*-PMMA, and P4VP-*b*-PI [26,27,28,29]. In these cases, these block segments are immiscible. To the best of our knowledge, there are few studies that investigate immiscible A-*b*-B with disordered C-*b*-D block copolymers to provide hierarchical self-assembled structures. In our previous work [39], we proposed immiscible PS-*b*-P4VP blending with miscible disordered PVPh-*b*-PMMA to form hierarchical self-assembled structures such as three lamellar or core–shell cylinder nanostructures. In this work, we propose another immiscible PS-*b*-PVPh diblock copolymer blend with disordered PEO-*b*-P4VP diblock copolymer, which is also mediated by using competitive hydrogen bonding strength to form the hierarchical self-assembled structures. 

## 2. Results and Discussion

### 2.1. The Preparation of PS-b-PVPh and PEO-b-P4VP Diblock Copolymers

**Scheme 1:**
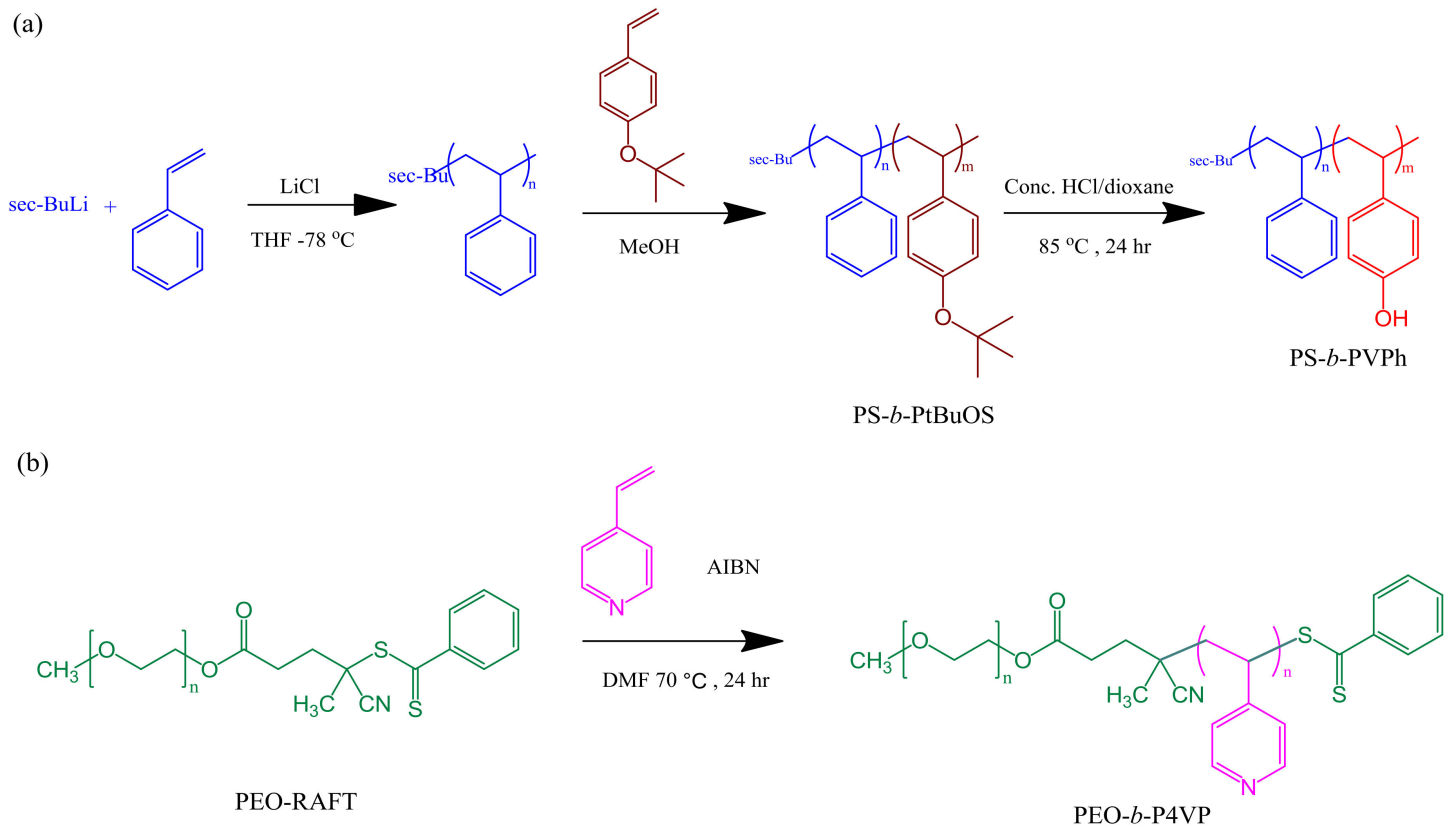
Synthesis of (**a**) PS-*b*-PVPh by sequential anionic living polymerization and (**b**) PEO-*b*-P4VP by RAFT polymerization.

**Figure 1:**
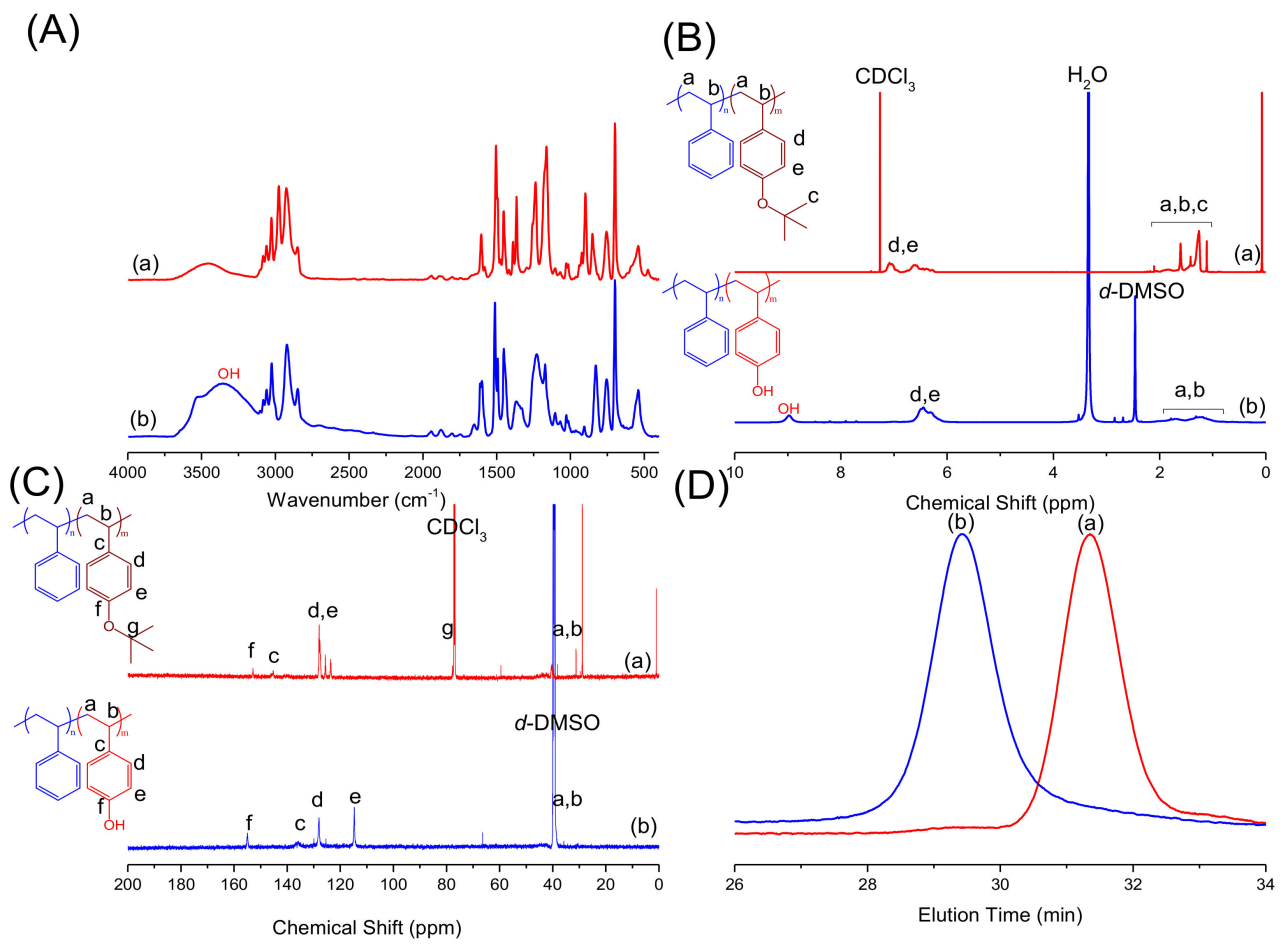
(**A**) FTIR, (**B**) ^1^H-, (**C**) ^13^C-NMR, and (**D**) GPC analyses of (a) PS-*b*-PtBuOS and (b) PS-*b*-PVPh.

In previous studies [11,13,40] we have proposed the preparation of PS-*b*-PVPh diblock copolymers using anionic polymerization and then performing a hydrolytic reaction to remove *tert*-butyl ether units from poly(*tert*-butoxystyrene) (PtBuOS, Scheme 1a). We observed the formation of phenolic OH units for PS-*b*-PVPh diblock copolymer based on the complete removal of the protective *tert*-butyl groups based on FTIR, NMR and GPC analyses. Firstly, the PS-*b*-PVPh spectrum with a broad band at 3360 cm^−1^ and a sharp absorption at 3530 cm^−1^, represents the self-association and free OH groups after deprotection as shown in Figure 1A(b). Secondly, the signal at 1.30 ppm in the ^1^H-NMR (Figure 1B(a)) and 78.0 ppm in the ^13^C-NMR (Figure 1C(a)) spectra of PS-*b*-PtBuOS diblock copolymer both correspond to the *tert*-butyl ether groups and both signals disappeared after the hydrolysis reaction, while a signal at 9.0 ppm corresponding to OH units of PVPh block appeared (Figure 1B(b)). In addition, the signal of the Cf–OH unit was shifted from 153.6 ppm (Figure 1C(a)) to 155.8 ppm (Figure 1C(b)), also indicating the complete hydrolysis. Finally, the GPC trace of PS-*b*-PVPh displays a higher molecular weight shift compared with PS-*b*-PtBuOS, probably due to the polymer-stationary phase interaction because of the hydrogen bonding interaction, as shown in Figure 1D. The number average molecular weight (*M*n) and polydispersity of PS_460_-*b*-PVPh_158_ were calculated as 63,000 g/mole and 1.04 based on Figure 1D(a).

**Figure 2:**
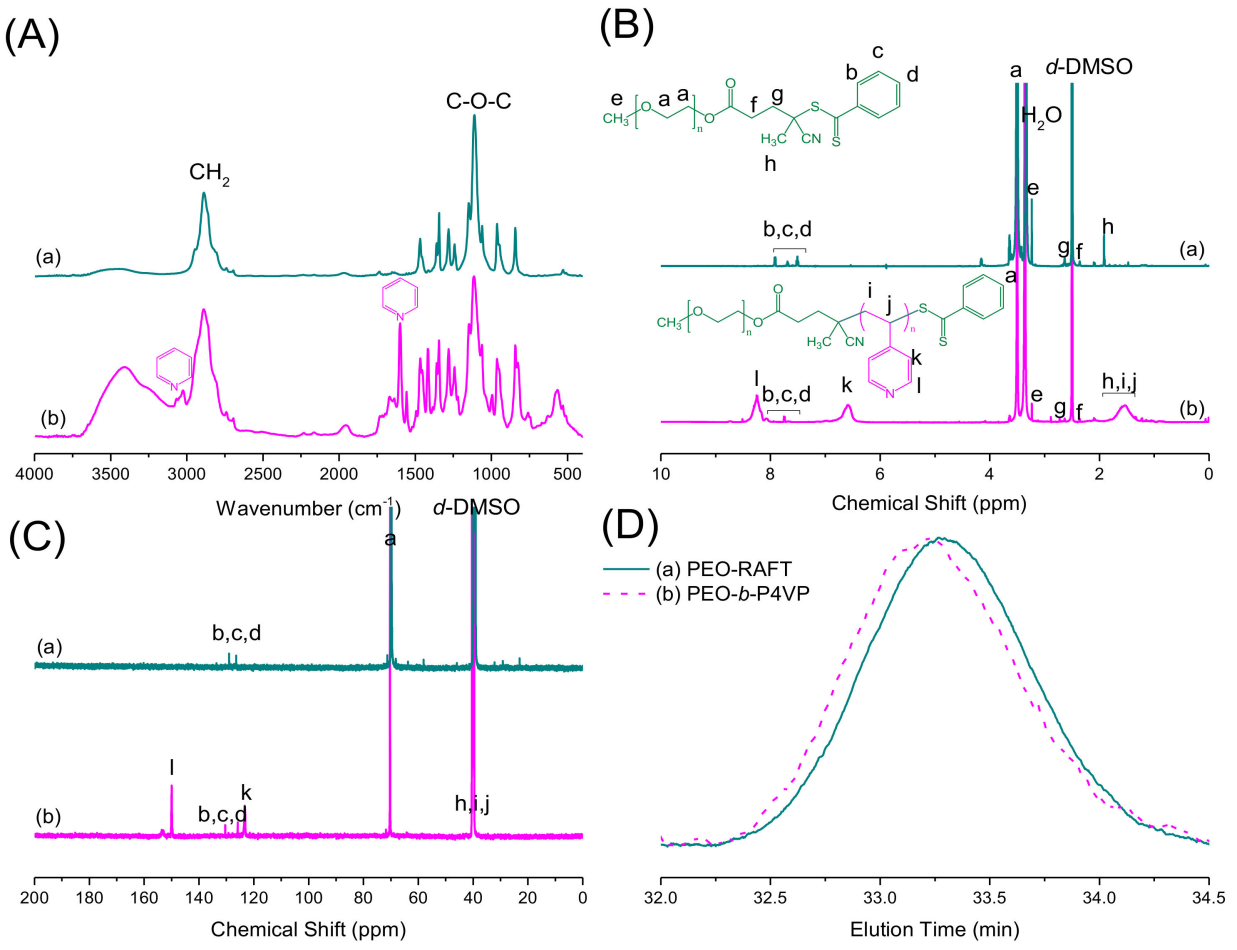
(**A**) FTIR, (**B**) ^1^H, (**C**) ^13^C-NMR, and (**D**) GPC analyses of (a) PEO-RAFT and (b) PEO-*b*-P4VP.

We also synthesized the PEO-*b*-P4VP diblock copolymer by RAFT polymerization as shown in Scheme 1b. Firstly, the spectrum of the PEO-RAFT macro-initiator displays a C-O-C stretching band at 1110 cm^−1^ (Figure 2A(a)) and the PEO-*b*-P4VP diblock copolymer features extra pyridine units at 993, 1598 and 3023 cm^−1^ as shown in Figure 2A(b). Furthermore, the CH_2_ unit of PEO segment was located at 3.50 ppm in the ^1^H-NMR spectrum and the other peak assignments of PEO-RAFT macro-initiator are displayed in Figure 2B(a).

After the RAFT polymerization to form PEO-*b*-P4VP diblock copolymer, signals at 6.59 and 8.25 ppm corresponding to the pyridine units appear, as shown in Figure 2B(b). In addition, the ^13^C-NMR spectrum of PEO-*b*-P4VP diblock copolymer is displayed in Figure 2C, where the CH_2_ unit of the PEO segment is located at 70.0 ppm (Figure 2C(a)) and the aromatic rings from the RAFT agent and the P4VP segment are distributed at 122~149 ppm (Figure 2C(b)). The GPC analyses (Figure 2D) of PEO-RAFT macro-initiator featured a mono-modal curve and narrow polydispersity, and after the RAFT polymerization, the signal was shifted to the lower retention time and also displayed a narrow polydispersity, implying the formation of PEO-*b*-P4VP diblock copolymer. We also could determine the number average molecular weight (Mn) and polydispersity of PEO_220_-*b*-P4VP_82_ as 18700 g/mole and 1.11 based on ^1^H-NMR and GPC analyses.

### 2.2. Self-Assembled Structure of PS-b-PVPh/PEO Diblock Copolymer/Homopolymer Mixtures

**Figure 3:**
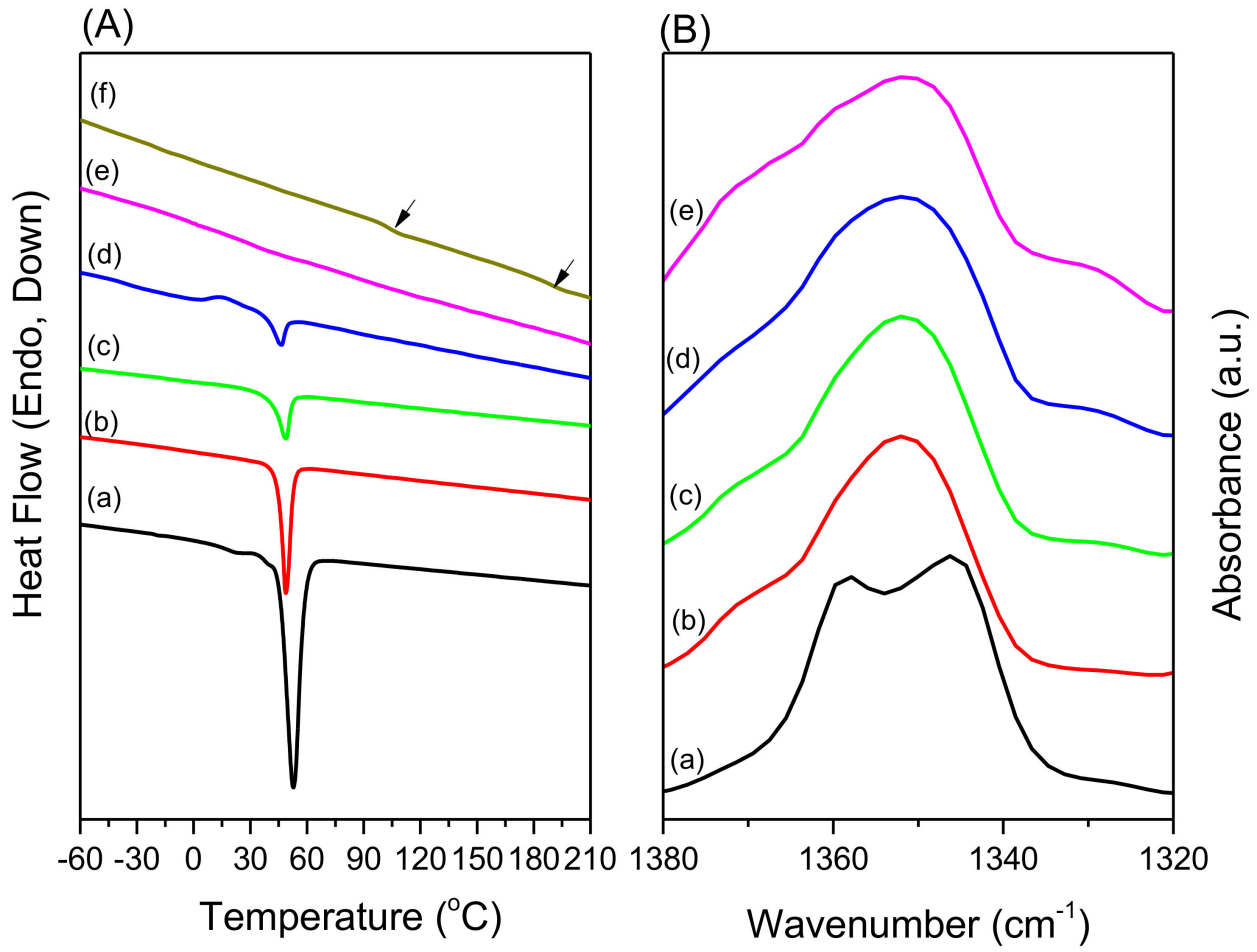
(**A**) DSC and (**B**) FTIR analyses of PS-*b*-PVPh/PEO blends with different PEO weight percent (a) 100, (b) 80, (c) 60, (d) 40, (e) 20, and (f) 0 wt.%.

To understand the self-assembly behavior and completive hydrogen bonding interactions of PS-*b*-PVPh/PEO-*b*-P4VP mixtures, we should understand the corresponding behavior of individual PS-*b*-PVPh/PEO and PS-*b*-PVPh/P4VP blends. In our previous studies [11,13], we have investigated the self-assembly structures of PS-*b*-PVPh/P4VP blends, which exhibit the wet-brush behavior featuring fully order-order morphological transition from lamellae, gyroid, cylindrical, and BCC spherical nanostructures. As a result, we firstly investigated the phase behavior of the PS_224_-*b*-PVPh_854_/PEO_220_ blend system. Figure 3 displays the second heating scan of the DSC thermograms (Figure 3A) and CH_2_ wagging vibration of the PEO segment based on FTIR analyses (Figure 3B) of the PS-*b*-PVPh/PEO blend system. Figure 3A(a) displays the melting temperature of pure PEO at 53 °C and pure PS-*b*-PVPh displays two *T_g_* values at 188 °C for the PVPh segment and 107 °C for the PS segment (Figure 1A(f)), suggesting the microphase separation of the PS-*b*-PVPh diblock copolymer. We also observe that the melting temperature (*T*_m_) and enthalpy of melting of PEO was decreased upon increasing the PS-*b*-PVPh concentrations due to the hydrogen bonding interaction of PVPh/PEO miscible phase for thermodynamic reasons and the morphology effect. The depression of PEO crystalline phase was also investigated by FTIR analyses as displayed in Figure 3B. 

The absorptions at 1343 and 1360 cm^−1^ were related to the PEO crystalline phase and these two absorptions disappeared and were replaced by a broad absorption at 1350 cm^−1^ due to the PEO amorphous phase upon increasing the PS-*b*-PVPh concentrations [41]. As a result, the PEO crystallization is inhibited and retarded upon increasing the amorphous PS-*b*-PVPh diblock copolymer. 

**Figure 4:**
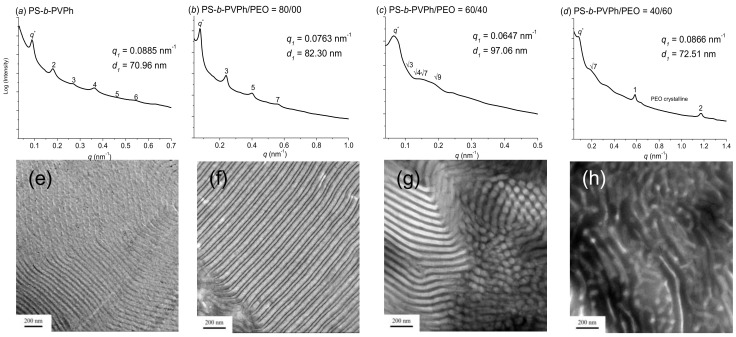
SAXS and TEM images of PS-*b*-PVPh/PEO blends of (**a**,**e**) 100/0, (**b**,**f**) 80/20, (**c**,**g**) 60/40, and (**d**,**h**) 40/60.

Figure 4 shows SAXS and TEM analyses for the PS-*b*-PVPh/PEO blend system with various PEO compositions at room temperature. Pure PS-*b*-PVPh displayed a lamellar structure long range order as displayed in Figure 4a from the scattering peak ratios of 1:2:3:4:5:6, which could be confirmed by the TEM image (Figure 4e). As the PEO composition was increased to 20 wt%, the lamellar structure still remained, with a scattering ratio of 1:3:5:7, which is also consistent with the TEM image (Figure 4f). Since the even-order peaks (such as 2, 4, and 6) almost disappeared, it implied that the PS volume fraction is close to 0.5 in this case (*f*_PS_^v^ = 0.51 at this blend composition). Furthermore, the first scattering peak was shifted to a relatively lower value, from *q* = 0.0885 nm^–1^ (*d* = 70.96 nm) to *q* = 0.0763 nm^−1^ (*d* = 82.30 nm), suggesting an increase of the block copolymer spacing. This phenomenon could also be explained by the fact that the PS block segment goes from an asymmetric to a symmetric composition, suggestion that the PEO segment could be dissolved in the PVPh block segment through intermolecular hydrogen bonding interactions and change the volume fraction of the block copolymer. Furthermore, the SAXS peak ratio was observed at 1:√3:√4:√7:√9, representing the hexagonally packed cylindrical nanostructure at 40 wt% PEO composition as displayed in Figure 4c, and confirmed by the TEM image (Figure 4g). The first scattering peak was further shifted to a lower *q* value (*q* = 0.0647 nm^–1^, *d* = 97.06 nm), also suggesting the increase of block copolymer spacing. However, it displayed wormlike or disordered micelle structures at 60 wt% PEO composition, where the first scatting peaks was observed and the broad peak ratio of √7, as displayed in Figure 4d, as confirmed by the TEM image (Figure 4h). Furthermore, we also observed a crystalline lamellar structure for peak ratio of 1:2 where the *q* value of (*q*_1_ = 0.58 nm^−1^, *d* = 10.82 nm, *q*_2_ = 1.16 nm^−1^, *d* = 5.41 nm) and thus the self-assembly structure from miscible PVPh/PEO phase also competed with the crystallization behavior of the PEO domain in this case. Overall, we could conclude that the PS-*b*-PVPh/PEO blends also display the wet-brush behavior where *K*_A_ = 280 of PVPh/PEO is stronger than the *K*_B_ = 66.8 of PVPh, which is consistent with our previous proposed scheme. However, it did not display a full order-order morphological transition, probably because of the relatively weaker intermolecular hydrogen bonding strength as compared with strong PVPh/P4VP blend system (*K*_A_ = 1200) [11,12,13,42,43,44,45].

### 2.3. Self-Assembled Structure of PS-b-PVPh/PEO-b-P4VP Diblock Copolymer Mixtures

**Scheme 2:**
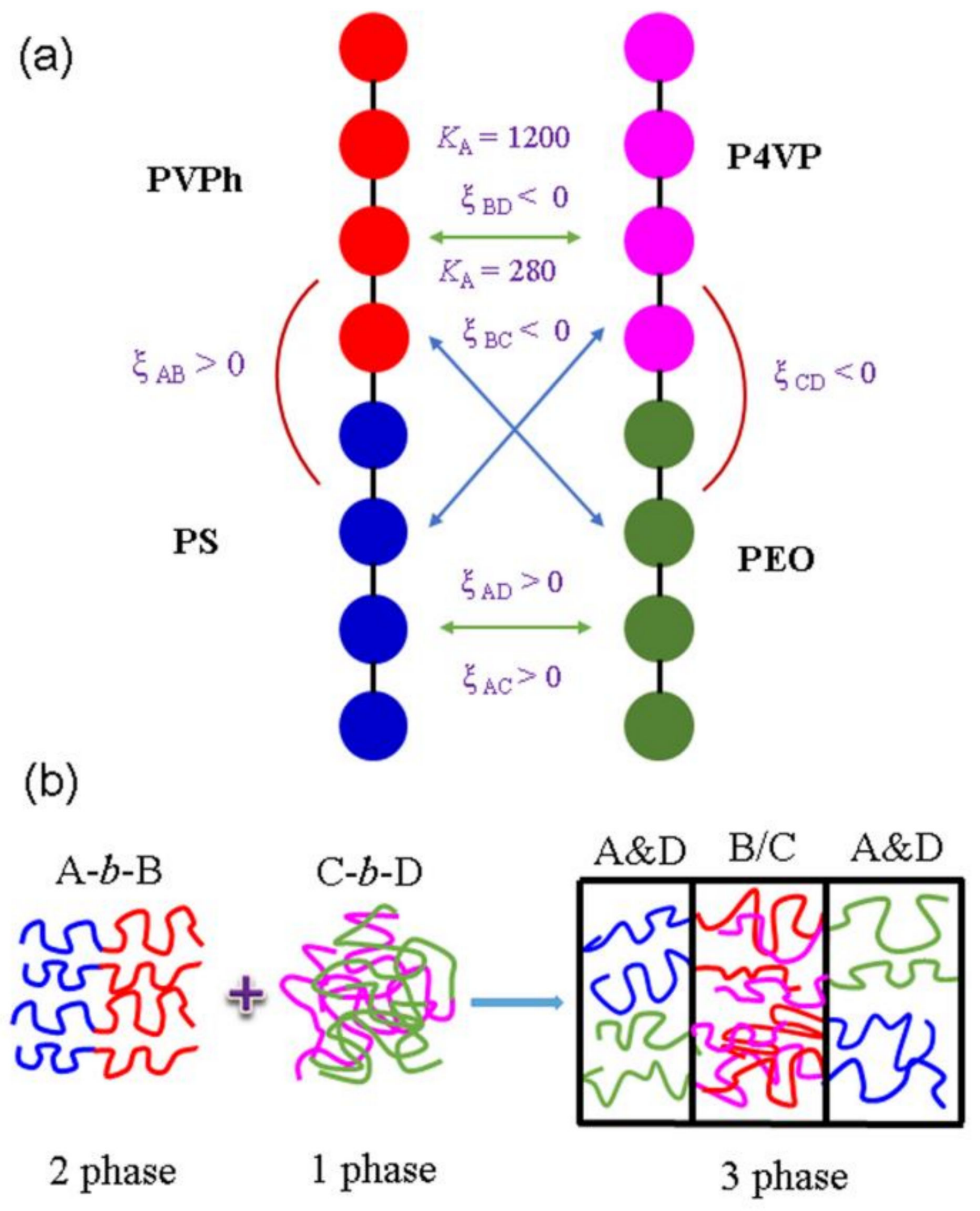
(**a**) Inter-association equilibrium constant and interaction parameters of PS-*b*-PVPh/PEO-*b*-P4VP mixtures and (**b**) cartoon representation of self-assembly nanostructures for A-*b*-B/C-*b*-D mixtures in this study.

In this study, we prepared two different diblock copolymers by using anionic living (PS_158_-*b*-PVPh_460_) and RAFT (PEO-*b*-P4VP) polymerizations with hydrogen bonded donor or acceptor groups as displayed in Scheme 1. The molecular weights, solubility parameters, molar volumes, equilibrium constants, and interaction parameters of each block segment are summarized in Table 1 and Scheme 2. 

Molecular volume, molecular weight, solubility parameter, self-association dimer (*K*_2_) and multimer (*K*_B_), and inter-association (*K*_A_) equilibrium constants were determined based on reference [44]. 

**Figure 5:**
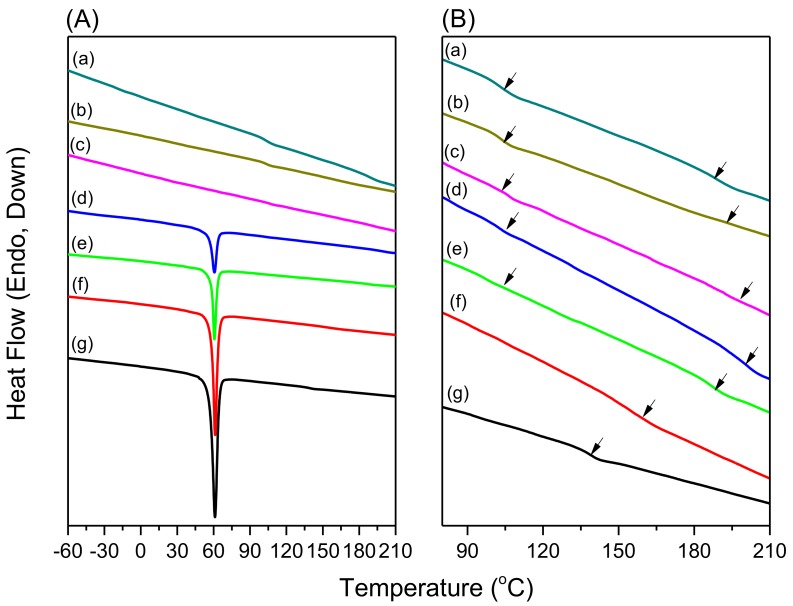
DSC analyses of PS-*b*-PVPh/PEO-*b*-P4VP mixtures of range from (**A**) −60~210 °C, and (**B**) 80~210 °C for (a) 100/0, (b) 90/10, (c) 70/30, (d) 50/50, (e) 30/70, (f) 10/90, and (g) 0/100.

As shown in Scheme 2, six different interaction parameters are expected in PS-*b*-PVPh/PEO-*b*-P4VP mixtures. Pure PS-*b*-PVPh block copolymers display the typical upper critical ordering transition (UCOT) phase behavior, implying that the microphase separation was occurred at lower temperature (Figure 4(a)) and become disordered structures at a higher temperature (order-to-disorder transition (ODT)) because of reduction the interaction parameter (χ) with the increase of temperature, which was observed for most diblock copolymer systems. However, Chen et al. have proposed that PEO-*b*-P4VP diblock copolymers possess a lower critical ordering transition (LCOT), which is similar to the lower critical solution temperature (LCST) in polymer blend systems [46]. This disorder-to-order transition (DOT) was rarely observed in diblock copolymer systems, implying that the PEO-*b*-P4VP diblock copolymer displays a disordered structure at lower temperature. As a result, three interaction parameters would be negative including PVPh/P4VP, PVPh/PEO, and PEO/P4VP miscible or disorder binary blends and the other three interaction parameters would be positive indicating that PS/PEO, PS/PVPh, and PS/P4VP are immiscible binary blends However, the *K*_A_ value of the PVPh/P4VP (1200) [42,43,44,45] is much greater than that of PVPh/PEO (*K*_A_ = 280) [44] indicating that the PVPh would tend to interact more with the P4VP segment than the PEO segment. Mediating this competitive hydrogen bonding strength with different blend compositions, the PEO block might possess microphase separation from disordered PEO-*b*-P4VP diblock copolymer during blending with PS-*b*-PVPh and then induce three phases (PS, PVPh/P4VP, and PEO phases) or even hierarchical self-assembled structures as displayed in Scheme 2b. DSC analysis is widely used for understanding the miscibility behavior of polymer blends. Figure 5A shows the heating thermograms of PS-*b*-PVPh/PEO-*b*-P4VP blends with various compositions based on DSC analyses from −60 °C to 210 °C. Similarly, pure PS-*b*-PVPh also displays two *T*_g_ values at 188 °C for PVPh segment and 105 °C for PS segment (Figure 5(a)) and pure PEO-*b*-P4VP diblock copolymer displays a *T*_m_ value at 61 °C for the PEO segment and a *T*_g_ value at 140 °C for the P4VP segment (Figure 5(f)). It was difficult to observe the *T*_g_ value for the PEO segment in this study because of the high crystallinity behavior of this diblock copolymer. Compared with the PS-*b*-PVPh/PEO blend system, the melting temperature of the PEO block segment in PEO-*b*-P4VP almost remained constant with the increase of PS-*b*-PVPh concentration (<70 wt%), indicating that the phenolic OH unit of PVPh did not interact with the ether units of PEO in PS-*b*-PVPh/PEO-*b*-P4VP mixtures at these compositions. Due to the high crystallinity behavior of PEO block segment, we expanded the DSC thermograms from 80 °C to 210 °C to avoid the melting behavior of the PEO segment.

As shown in Figure 5B. Clearly, we found two *T*_g_ values for all diblock copolymer mixture compositions, suggesting that microphase separation occurred for these diblock copolymer mixtures. The lower *T*_g_ values (105–106 °C) corresponded to the PS segment, while the higher *T*_g_ values (161–202 °C) are due to the miscible PVPh/P4VP domain through strong hydrogen bonding interactions, appear higher than both individual homopolymers at lower PEO-*b*-P4VP blend compositions. Furthermore, these *T*_g_ values were also higher than the binary homopolymer blend of PVPh/P4VP = 1/1 from DMF solution (*T*_g_ = 190 °C) presumably due to the nanoconfinement effect from microphase separation of the diblock copolymer mixtures in this study. As a result, we could observe the higher *T*_g_ behavior at 161–202 °C from the miscible PVPh/P4VP domain, the lower *T*_g_ values at 105–106 °C from PS segment, and a melting temperature at ca. 60 °C for the PEO segment, indicating that at least three phases existed for the PS, PVPh/P4VP, and PEO domains in this PS-*b*-PVPh/PEO-*b*-P4VP blend. 

**Figure 6:**
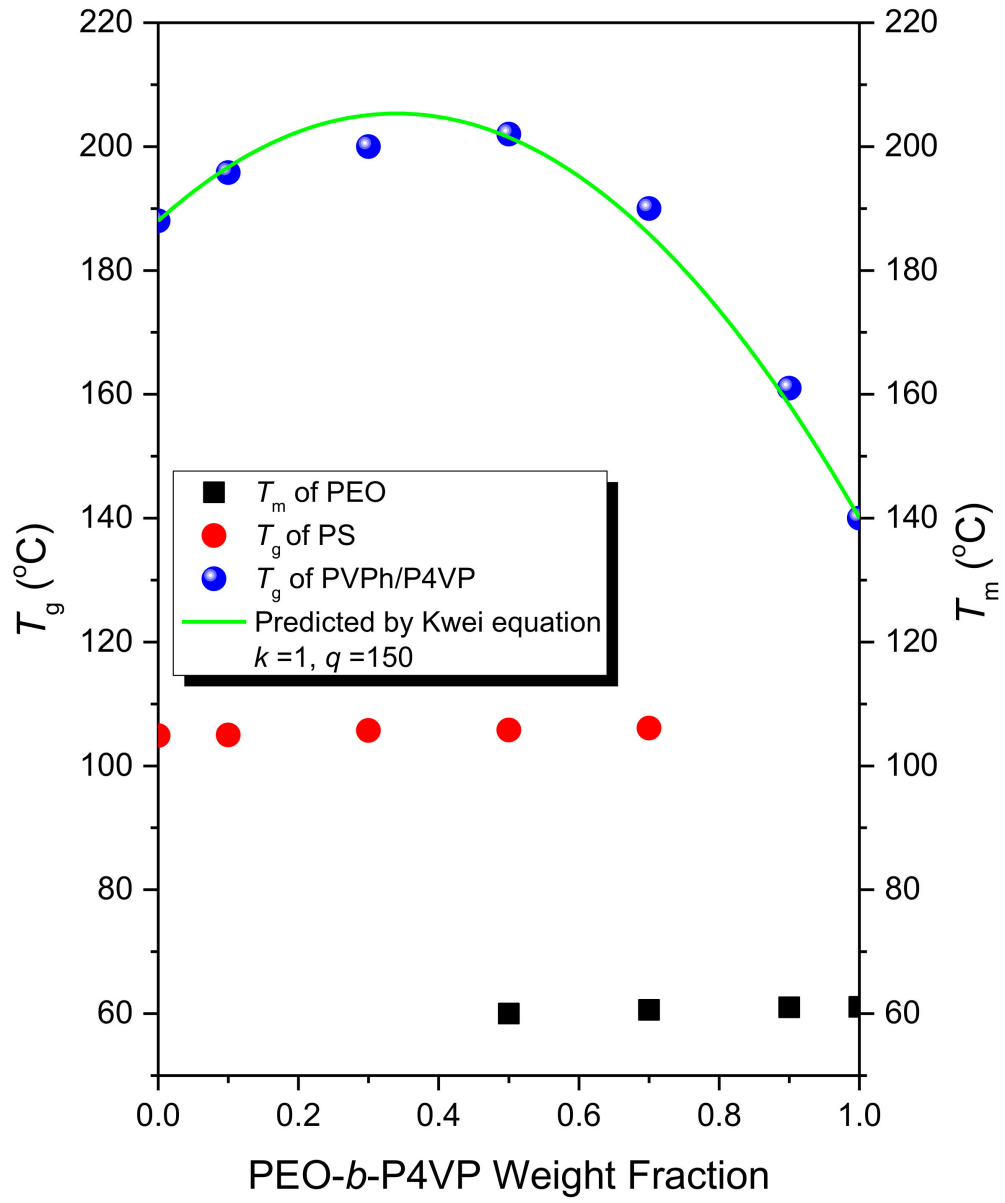
*T*_g_ behavior of PS and PVPh/P4VP domain (also predicted by Kwei equation), and *T*_m_ behavior of PEO segment of PS-*b*-PVPh/PEO-*b*-P4VP mixtures.

Figure 6 summarizes *T*_g_ behavior of the PS domain and PVPh/P4VP domain and *T*_m_ behavior of the PEO domain for PS-*b*-PVPh/PEO-*b*-P4VP blends. Clearly, the *T*_g_ value of PS domain and *T*_m_ value of PEO domain almost did not change with the various blend compositions; however, the *T*_g_ value of PVPh/P4VP domain displayed positive deviations based on the linear rule and these values could be predicted by the Kwei equation for strong hydrogen bonded blend systems [47]:

(1) $$T_{g}=\frac{W_{1}T_{g1\;}+kW_{2}T_{g2}}{W_{1}+kW_{2}}+qW_{1}W_{2}$$

where *T*_gi_ represents the glass transition temperature of each segment, *W_i_* represents each weight fraction of block segment; *k* and *q* are due to the fitting constant. We can determine the *k* and *q* values of 1 and 150, which represents the strong hydrogen bonding interaction in this work.

**Figure 7:**
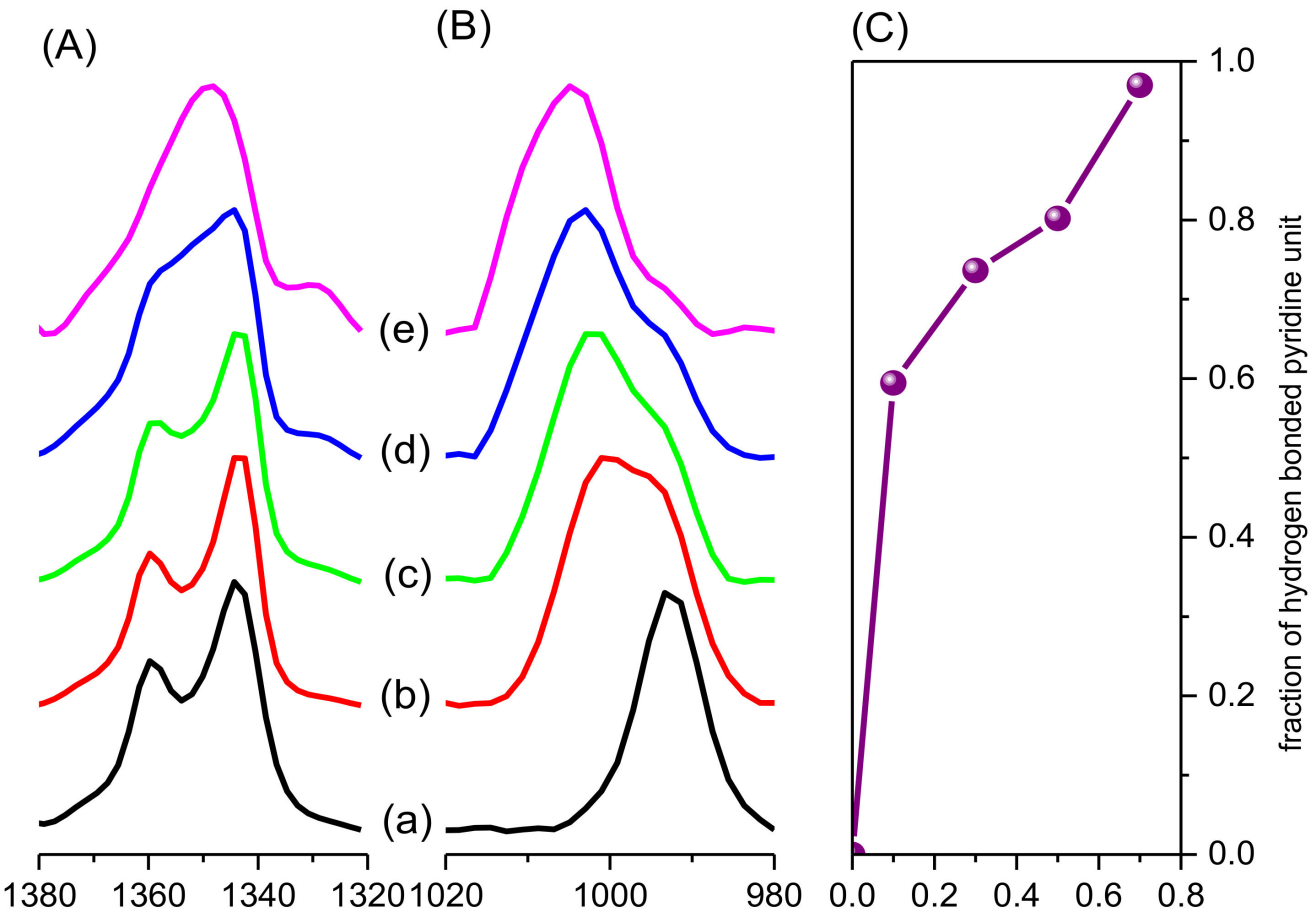
FTIR analyses of PS-*b*-PVPh/PEO-b-P4VP mixture of (**A**) CH_2_ wagging, (**B**) pyridine vibration of (a) 0/100, (b) 10/90, (c) 30/70, (d) 50/50, (e) 70/30, and (**C**) fraction of hydrogen bonded pyridine unit with different PS-*b*-PVPh weight fractions.

We used FTIR spectroscopy to investigate the intermolecular hydrogen bonding interaction and crystallization behavior of the PS-*b*-PVPh/PEO-*b*-P4VP diblock copolymer mixture as displayed in Figure 7. Figure 7A displays the FTIR spectra of pure PEO-*b*-P4VP for various PS-*b*-PVPh weight compositions as inferred from the 1380 to 1320 cm^−1^ region due to the CH_2_ wagging of the PEO segment. 

**Figure 8:**
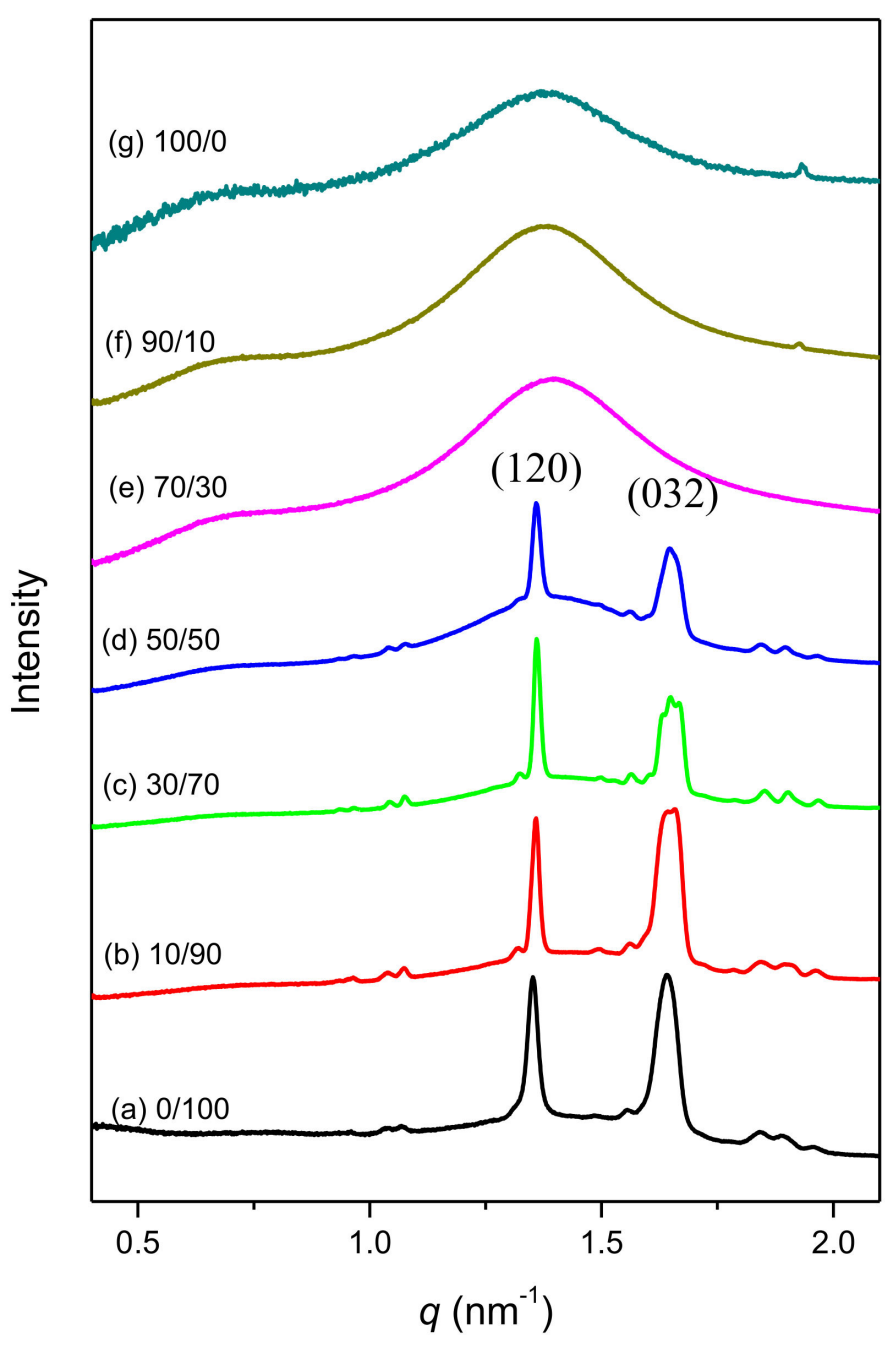
WAXD analyses of PS-*b*-PVPh/PEO-*b*-P4VP mixture at room temperature of (a) 0/100, (b) 10/90, (c) 30/70, (d) 50/50, (e) 70/30, (f) 90/10, and (g) 100/0.

Similar to Figure 3B, the absorptions at 1343 and 1360 cm^−1^ represent the PEO crystalline phase and these two absorptions disappear and were replaced by a broad absorption at 1350 cm^−1^ due to the PEO amorphous phase at 70 wt% of PS-*b*-PVPh concentrations, which is consistent with the DSC analyses where the melting peak disappeared for this blend composition. The result is also consistent with the WAXD analyses as displayed in Figure 8. Two strong (120)_PEO_ and (032)_PEO_ diffraction peaks for pure PEO-*b*-P4VP diblock copolymer were observed in Figure 8(a) and they disappeared at 70 wt% of PS-*b*-PVPh concentration. Furthermore, Figure 7B exhibits the absorption at 993 cm^–1^ for the free pyridine units of the P4VP segment and a new absorption at 1005 cm^−1^ was observed for the hydrogen bonded pyridine unit in the PVPh/P4VP domain. We determined the fraction hydrogen bonded pyridine units based on the digital subtraction at 1013 cm^−1^ due to the PVPh unit by considering the molar faction of PVPh block and we also observed that the value of fraction of hydrogen bonded pyridine unit was increased with the increase of PS-*b*-PVPh concentration as displayed in Figure 7C. 

**Figure 9:**
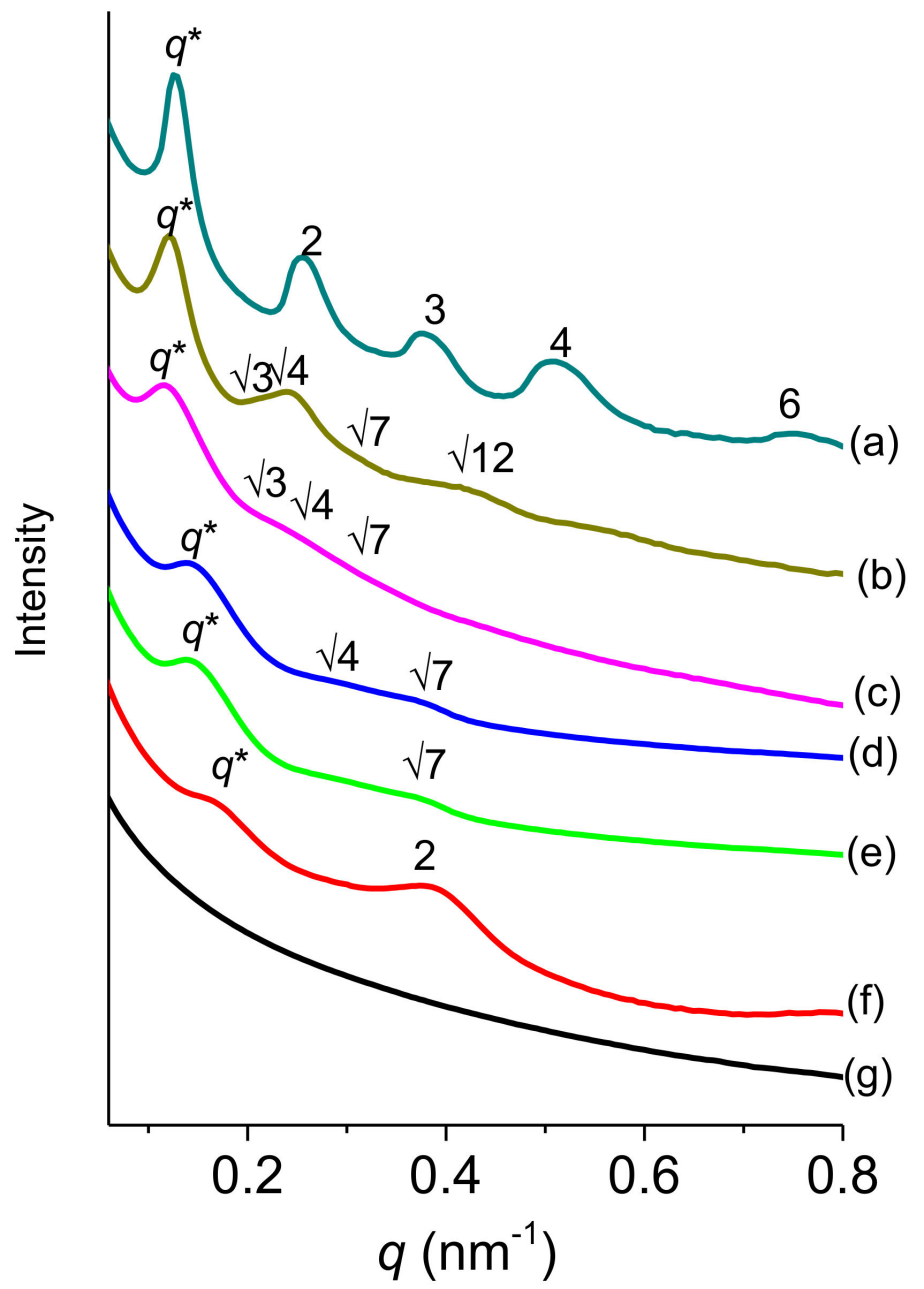
SAXS analyses of PS-*b*-PVPh/PEO-*b*-P4VP mixture at room temperature of (a) 0/100, (b) 10/90, (c) 30/70, (d) 50/50, (e) 70/30, (f) 90/10, and (g) 100/0.

**Figure 10:**
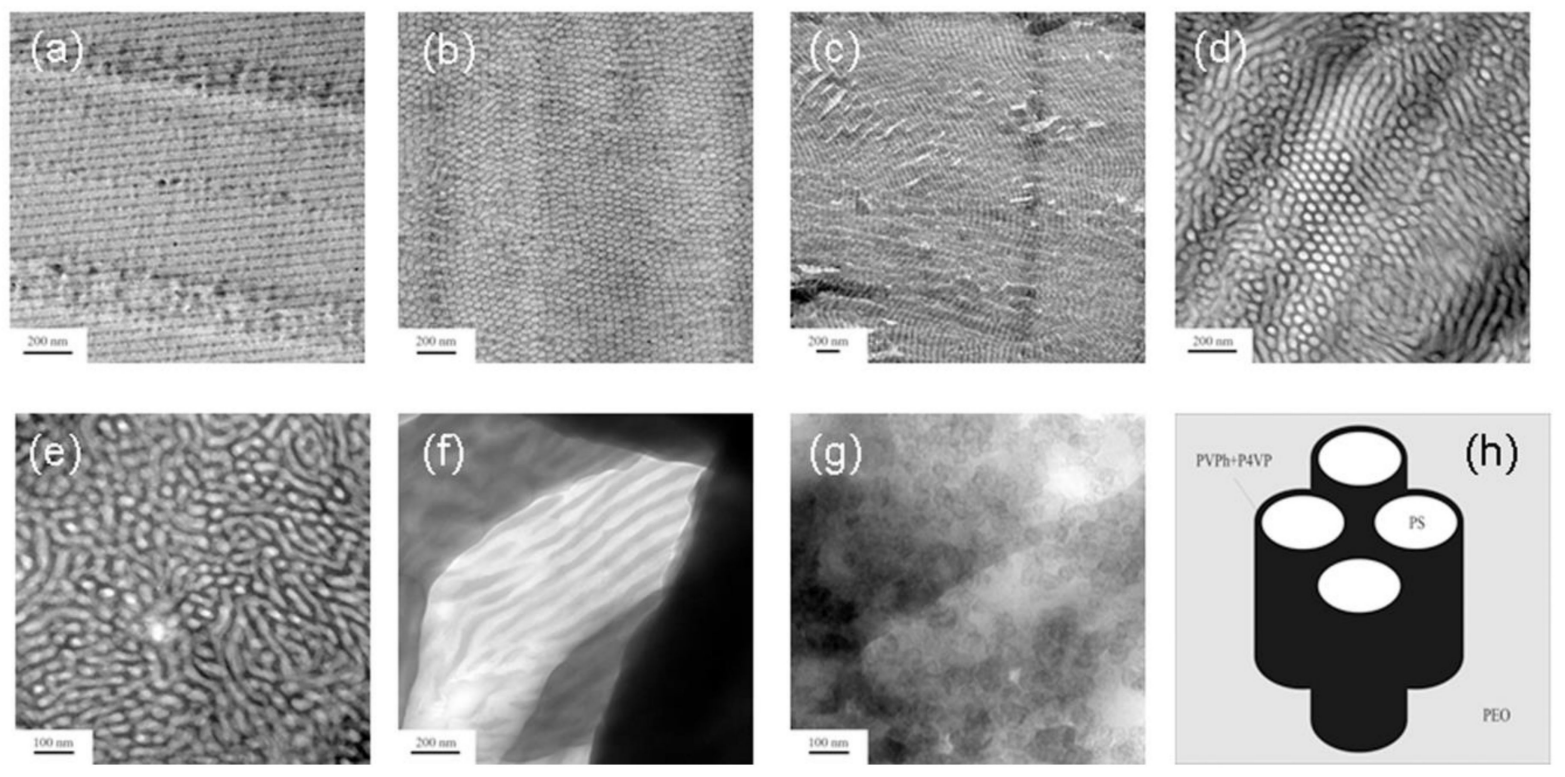
TEM images staining with I_2_ of PS-*b*-PVPh/PEO-*b*-P4VP mixture at room temperature of (**a**) 100/0, (**b**) 90/10, (**c**) 70/30, (**d**) 50/50, (**e**) 30/70, (**f**) 10/90, (**g**) 0/100, (**h**) cartoon representation of core-shell cylinder structure of PS, PVPh/P4VP, and PEO domains.

Figure 9 displays SAXS analyses for PS-*b*-PVPh/PEO-*b*-P4VP blends with different compositions at room temperature. Pure PS-*b*-PVPh as displayed in Figure 9A-(a) also shows the long range order of a lamellar structure based on the scattering peak ratio of 1:2:3:4:6 and the corresponding *d*-spacing lamellar structure is 50.2 nm from the first peak position at q* = 0.125 nm^−1^. The TEM image as displayed in Figure 10a also reveals that the pure PS-*b*-PVPh shows a lamellar structure, which is consistent with the SAXS pattern. In addition, pure PEO-*b*-P4VP diblock copolymer displays a disordered structure at lower temperature as shown in Figure 9(g) without any peak, as also confirmed by the TEM image (Figure 10g). 

As the PEO-*b*-P4VP concentration increases to 10 wt%, the SAXS pattern displays a highly ordered cylindrical structure with a scattering ratio of 1:√3:√4:√7:√12, as also consistent with the TEM analysis (Figure 10b) and the first scattering peak was shifted slightly to a *q* value at 0.121 nm^−1^ (*d* = 51.9 nm). Further increasing the PEO-*b*-P4VP concentrations to 30 or 50 wt% also results in cylindrical structures based on the peak ratios of 1:√3:√4:√7 as shown in Figure 9c,d and were also consistent with the TEM images in Figure 10c,d. The first scattering peaks appeared at *q* values of 0.117 nm^–1^ (*d* = 53.67 nm) at 30 wt% PEO-*b*-P4VP and 0.137 nm^–1^ (*d* = 45.83 nm) at 50 wt% PEO-*b*-P4VP. Clearly, the addition of the PEO-*b*-P4VP diblock copolymer could induce the order-order transition from lamellae to cylindrical structures featuring the wet-brush behavior. Upon further increasing the PEO-*b*-P4VP concentration to 70 wt%, a disordered wormlike structure with a peak ratio of 1:√7 is displayed as shown in Figure 9(e), consistent by the TEM image (Figure 10e). As the PEO-*b*-P4VP concentration increases to 90 wt%, it displays a lamellar structure with a peak ratio of 1:2 as displayed in Figure 9(f), also confirmed by the TEM image (Figure 10f). The lamellar structure may come from the crystallization behavior from the PEO block segment as displayed in the DSC, FTIR, and WAXD analyses. Since the driving force of PEO crystallization behavior may be favored more than the microphase separation of block segments thus the crystalline lamellar structure or short range order micellar structure are observed at relative higher PEO-*b*-P4VP concentrations. Based on these results, although the PS-*b*-PVPh/P4VP system could exhibit a fully order–order transition from lamellae to gyroid to cylinder and finally to BCC spheres, the PS-*b*-PVPh/PEO-*b*-P4VP mixtures only display the order–order transition from lamellae to hexagonally packed cylinders. The relatively weaker hydrogen bonding strength of the PEO segment compared with the P4VP segment and the crystallization behavior of the PEO segment all inhibit the fully order-order transition for PS-*b*-PVPh/PEO-*b*-P4VP mixtures in this study.

**Figure 11:**
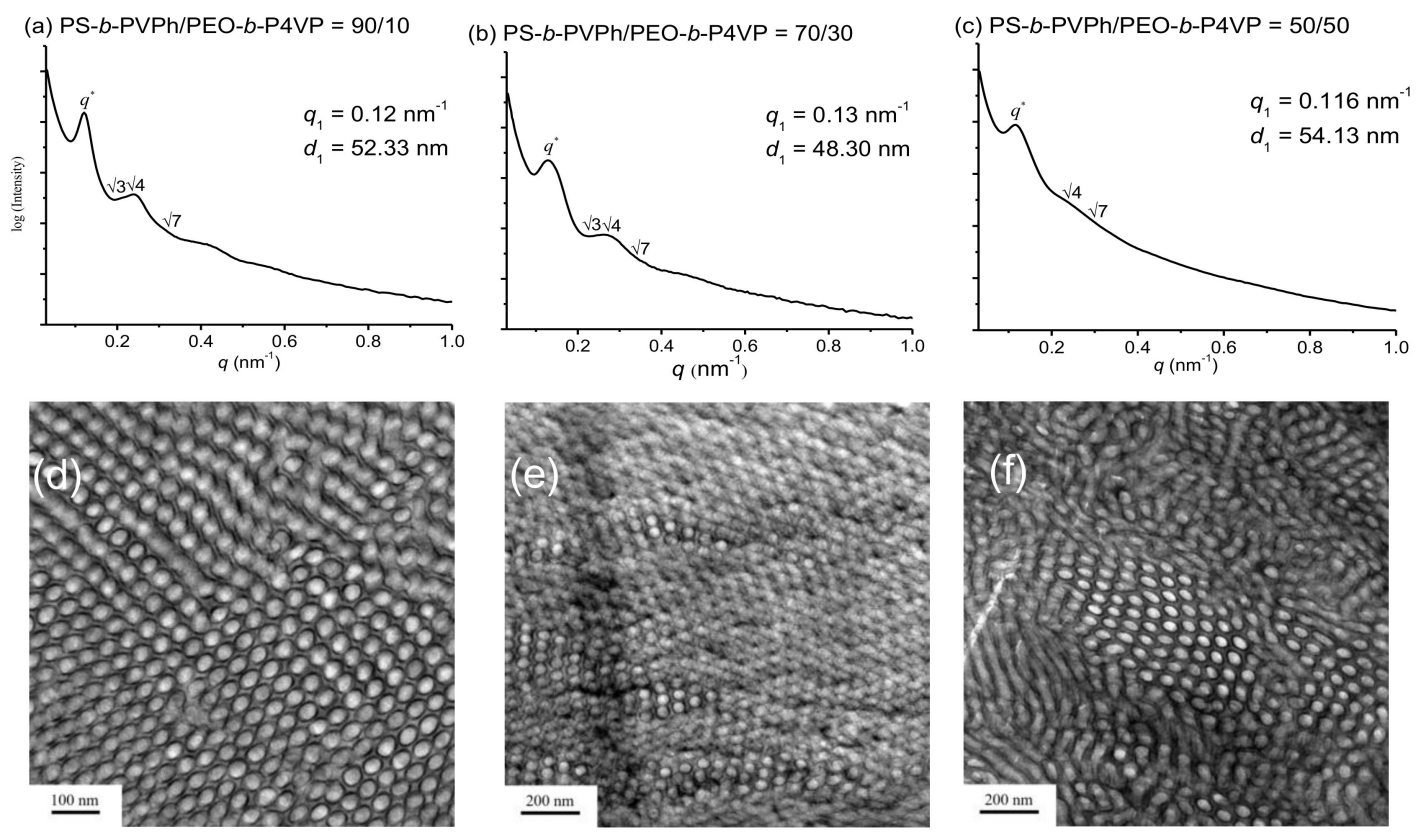
SAXS analyses and TEM images staining with I_2_ and RuO_4_ of PS-*b*-PVPh/PEO-*b*-P4VP mixture at room temperature of (**a**,**d**) 90/10, (**b**,**e**) 70/30, and (**c**,**f**) 50/50.

However, the advantage of PS-*b*-PVPh/PEO-*b*-P4VP mixture system is that it could exhibit three-phase behavior compared with PS-*b*-PVPh/PEO or PS-*b*-PVPh/P4VP diblock copolymer/ homopolymer blends. Here, we used RuO_4_ for further staining the PS-*b*-PVPh/PEO-*b*-P4VP mixtures. TEM images determined after staining with I_2_ could reveal the P4VP domains and further staining with RuO_4_ could provide the PEO domains in this diblock copolymer mixture. Figure 11d,f display TEM images for PS-*b*-PVPh/PEO-*b*-P4VP = 90/10, 70/30, and 50/50 determined after staining with both I_2_ and RuO_4_. Clearly, the PVPh/P4VP domain appears dark; the PS domain is white, and the PEO domain appears gray as displayed in Figure 10h. Since the *K*_A_ for PVPh/P4VP complex is much larger than that of PVPh/PEO, the PEO segment may be excluded from the disordered PEO-*b*-P4VP block copolymer and thus induce microphase separation. In addition, due to the intrinsic immiscibility behavior of the PEO and PS segments, the three phases of PS-*b*-PVPh/PEO-*b*-P4VP blends as expected show PS, PVPh/P4VP, and PEO domains as seen in Figure 10h.

## 3. Conclusions

We have successfully prepared PS-*b*-PVPh and PEO-*b*-P4VP by sequential anionic living and RAFT polymerizations according to FTIR, NMR and GPC analyses, respectively. Since the *K*_A_ value and hydrogen bonding strength of the PVPh/P4VP domain is much greater than those of the PVPh/PEO domain thus the PEO block segment was excluded from the disordered PEO-*b*-P4VP and then undergoes microphase separation. In addition, due to the intrinsic immiscible behavior of the PEO and PS block segments, the PS-*b*-PVPh/PEO-*b*-P4VP blends possesses a hierarchical self-assembled behavior as core–shell cylindrical structures by taking advantage of competitive hydrogen bonding and Δ*K* effect in this study.

## 4. Experimental Section

### 4.1. Materials

4-Vinylpyridine (4VP, 95%, Acros, Ward Hill, MA, USA) was distilled from CaH_2_. DMF was distilled from CaH_2_ after the heating under reflux for 4 h under N_2_. Poly(ethylene glycol) 4-cyano-4-(phenylcarbonothioylthio) pentanoate (PEO-SC(S)Ph) (*M*_n_ = 10000 g/mol) was purchased from Aldrich (St. Louis, MO, USA). The synthesis of PS-*b*-PVPh diblock copolymer was performed according to our previous works [11,13,40]

### 4.2. The Synthesis of PEO-b-P4VP Diblock Copolymer by Using RAFT Polymerization

AIBN (23 mg) and PEO-SC(S)Ph (0.3042 g) were mixed in DMF (8.6 mL). After three cycles of freeze-vacuum-thaw degassing process, then 4VP monomer (0.4 mL 3.7 mmol) was added and the solution was immersed in an oil bath at 70 °C under a N_2_ atmosphere for 24 h. After cooling to 25 °C, the reaction was stopped and the mixture dropped into diethyl ether. The precipitate was filtered and dried under vacuum overnight (yield: 66 wt%).

### 4.3. PS-b-PVPh/PEO-b-P4VP Blends

Different PS-*b*-PVPh/PEO-*b*-P4VP blend compositions are prepared by using solution casting, which are firstly dissolved in DMF since these four block segments are all soluble in DMF (5 wt%). The block copolymer mixtures were stirred for 48 h at 25 °C and were evaporated at 90 °C slowly for 48 h and dried under the vacuum at 120 °C for 96 h to remove the residual DMF solvent. 

### 4.4. Characterizations

FTIR spectroscopy of these blend samples were measured through the conventional KBr disk method and a Tensor 27 spectrophotometer (Bruker, Billerica, MA, USA) with 32 scans and a spectral resolution of 4 cm^−1^ were used. Molecular weights and their corresponding polydispersities of block copolymers were determined through gel permeation chromatography (GPC) using a Waters 510 HPLC system (GPC, Waters, Taipei, Taiwan) and DMF was used as the eluent. The calibration curve was constructed by PS standard with the combinations of refractive index, UV, viscosity, and light scattering detectors. NMR spectra were recorded on an AM 500 spectrometer (Bruker, Billerica, MA, USA) using CDCl_3_ or *d*-DMSO as *d*-solvents. Thermal properties are measured using a TA Q-200 instrument (TA Instrument, New Castle, DE, USA) from room temperature to 260 °C at 20 °C/min heating rate. SAXS analyses of these diblock copolymer mixtures were recorded using the BL23A1 (*λ* = 1.1273 Å) beamline (National Synchrotron Radiation Research Center, NSRRC), Taiwan. In general, blend specimens are sealed between Kapton films and determined at room temperature. TEM analyses of blend samples were performed using a JEOL 2100 microscope (Tokyo, Japan), which was operated at 200 kV. The diblock copolymer mixture films were cut into ultrathin sections by an ultra-cut microtome (Leica, Taipei, Taiwan) equipped with a diamond knife. The TEM images were determined after staining with I_2_ to exhibit the P4VP segments and further stained with RuO_4_ to find the PVPh and PEO domains. Thus, the PVPh/P4VP domains appear dark, the PEO domains appear gray, while the PS domains appear white in this diblock copolymer mixture.

## Figures and Tables

**Table 1:** Molecular volume, molecular weight, solubility parameter, and equilibrium constants of various polymer segments used in this work.

Polymer	Molar Volume	Molecular Weight	Solubility Parameter	Equilibrium Constants		
	(mL/mol)	(g/mol)	((cal/mL)^1/2^)	*K*_2_	*K*_B_	*K*_A_
PVPh	82.3	120.1	11.0	21.0	66.8	
PS	93.9	104.1	9.5	–	–	–
PEO	38.1	44.1	9.40	–	–	280
P4VP	84.9	105.1	10.8	–	–	1200
